# Factors associated with post-COVID-19 health conditions at a rehabilitation center: a cross-sectional study, Paraíba, Brazil, 2024

**DOI:** 10.1590/S2237-96222026v35e20250305.en

**Published:** 2026-04-20

**Authors:** Karla Karolline Barreto Cardins, Ísis de Siqueira Silva, Amanda Soares, Lara Maria Alves de Carvalho, José Mateus Bezerra da Graça, Cláudia Helena Soares de Morais Freitas

**Affiliations:** 1 Universidade Federal de Campina Grande, Unidade Acadêmica de Enfermagem, Cajazeiras, PB, Brazil; 2 Universidade Federal do Rio Grande do Norte, Natal, RN, Brazil; 3 Universidade Federal do Rio Grande do Norte, Faculdade de Ciências da Saúde do Trairi, Trairi, RN, Brazil; 4 Universidade Federal do Rio Grande do Norte, Programa de Pós-Graduação em Saúde Coletiva, Santa Cruz, RN, Brazil; 5 Universidade Federal da Paraíba, Departamento de Clínica e Odontologia Social, João Pessoa, PB, Brazil

**Keywords:** Keywords: Post-Acute COVID-19 Syndrome, Health Care, Rehabilitation Services, Signs and Symptoms, Cross-Sectional Studies, Síndrome Post Agudo de COVID-19, Atención de Salud, Servicios de Rehabilitación, Signos y Síntomas, Estudios Transversales

## Abstract

**Objective:**

To characterize the profile of people with post-COVID-19 conditions and factors associated with treatment outcome at a rehabilitation center in Campina Grande, Paraíba state, Brazil.

**Methods:**

This was a cross-sectional study, based on the analysis of 398 medical records of public health service users treated at a post-COVID rehabilitation center. Sociodemographic, clinical and care variables were assessed using prevalence ratios (PR) with 95% confidence intervals (95%CI) and a 5% significance level. The outcome of interest was the treatment result, categorized as hospital discharge or dropout.

**Results:**

The analysis of the 398 medical records revealed a predominance of females (62.6%) and adults in the 18-59 age group (63.3%). Treatment dropout was more frequent among females than among males (PR 1.68; 95%CI 1.11; 2.53; p-value 0.012). Hospitalized patients had lower risk of treatment dropout (PR 0.48; 95%CI 0.31; 0.74; p-value 0.001), and those who received oxygen therapy also had lower risk of treatment dropout (PR 0.50; 95%CI 0.31; 0.82; p-value 0.004). Of the 398 users analyzed, 282 (78.1%) were discharged from the rehabilitation center and 79 (21.9%) dropped out of treatment.

**Conclusion:**

Being female was associated with higher dropout rates, while hospitalization and oxygen therapy acted as protective factors for adherence. These findings emphasize the need for targeted follow-up strategies capable of reducing losses to follow-up and promoting greater effectiveness in post-COVID-19 rehabilitation within the Brazilian Unified Health System (Sistema Único de Saúde, SUS).

## Resumen

**Objetivo:**

Caracterizar el perfil de las personas con afecciones post-COVID-19 y los factores asociados con el resultado del tratamiento en un centro de rehabilitación en Campina Grande, estado de Paraíba, Brasil.

**Métodos:**

Estudio transversal basado en el análisis de 398 registros médicos de usuarios atendidos en un centro de rehabilitación post-COVID. Se evaluaron variables sociodemográficas, clínicas y de atención mediante la razón de prevalencia (RP) con un intervalo de confianza del 95% (IC95%) y un nivel de significancia del 5%. El resultado de interés fue el resultado del tratamiento, categorizado como alta hospitalaria o abandono.

**Resultados:**

El análisis de las 398 historias clínicas reveló un predominio del sexo femenino (62,6%) y del grupo de edad adulta de 18 a 59 años (63,3%). El abandono del tratamiento fue más frecuente en mujeres que en hombres (RP 1,68; IC95% 1,11; 2,53; valor-p 0,012). Los pacientes hospitalizados presentaron un menor riesgo de abandono del tratamiento (RP 0,48; IC95% 0,31; 0,74; valor-p 0,001), y quienes recibieron oxigenoterapia también presentaron un menor riesgo de abandono del tratamiento (RP 0,50; IC95% 0,31; 0,82; valor-p 0,004). De los 398 usuarios analizados, 282 (78,1 %) recibieron el alta hospitalaria y 79 (21,9 %) abandonaron el tratamiento.

**Conclusión:**

El sexo femenino se asoció con mayores tasas de abandono, mientras que la hospitalización y la oxigenoterapia actuaron como factores protectores para la adherencia. Estos hallazgos refuerzan la necesidad de estrategias de seguimiento específicas capaces de reducir las pérdidas de seguimiento y promover una mayor efectividad en la rehabilitación pos-COVID-19 en el Sistema Único de Salud (SUS).

**Ethical aspects**

This research respected ethical principles, having obtained the following approval data:

Research ethics committee: Universidade Estadual da Paraíba

Opinion number: 6,180,794

Approval date: 13/7/2023

Certificate of submission for ethical appraisal: 69833323.3.0000.5187

Informed consent form: Dismissed.

## Introduction 

In January 2020, the World Health Organization recognized COVID-19 as a public health emergency, given the rapid global spread of SARS-CoV-2 (1). Infection can range from asymptomatic and mild cases to moderate, severe and critical forms requiring hospitalization (1). Consequently, the pandemic overburdened health systems and caused increased morbidity and mortality, as well as significant repercussions on the mental health of the population, associated with stress, isolation and social uncertainties (2).

Even after the acute phase of the disease, a certain amount of people remained symptomatic for weeks or months afterwards. In response, the World Health Organization adopted the term “post-COVID syndrome”, characterized by the persistence of symptoms for more than three months, without being explained by other pre-existing conditions (3). Complementarily, the Brazilian Ministry of Health employed the term “post-COVID-19 conditions”, defined as signs, symptoms and/or conditions that persist or appear after four weeks of initial infection, which may evolve in a fluctuating manner, with worsening, relapses or serious complications, even months after infection (1).

This emerging scenario has imposed new challenges on healthcare networks and required reorganization of services and care strategies. In December 2021, the Brazilian Unified Health System (*Sistema Único de Saúde*) incorporated specific procedures for post-COVID rehabilitation, funded by the Strategic Actions and Compensation Fund (4). However, in the absence of consistent federal responses, several states and municipalities took the lead, creating their own treatment and rehabilitation initiatives (5,6).

Primary health care has played a central role in the assessment and initial management of people with post-COVID conditions, coordinating referral and counter-referral flows with health care networks. Some municipalities have organized specialized rehabilitation services to meet this growing demand (7). In this way, it becomes essential to know the profile of people seeking these services and the factors associated with the evolution of treatment, since such knowledge can support the organization of care, optimize resources, guide evidence-based clinical practices and strengthen public policies.

The objective of this study was to characterize the profile of people with post-COVID-19 conditions between January 2021 and December 2023, and factors associated with treatment outcomes at a rehabilitation center in Campina Grande, Paraíba state, Brazil.

## Methods 

### Design and setting 

This is a cross-sectional study, developed from analysis of electronic medical records of people served by the Campina Grande Municipal SuperAR Program. At the time of the study, the service had regional coverage and received both people referred to it and also spontaneous demand. Care was provided under the Brazilian Unified Health System. The data collection period covered the months of September to December 2023.

### Participants and eligibility criteria

The data for this research came from the medical records of users served by the program at the SuperAR Post-COVID Rehabilitation Center. The records included in the study were those of individuals who received assistance under the SuperAR Program with confirmed diagnosis of prior COVID-19 infection, along with accessible records relating to individuals who received treatment (respiratory physiotherapy, motor therapy, acupuncture and neurology) for post-COVID-19 symptoms between January 2021 and December 2023.

Records of psychological care were excluded because they were archived in a separate location, inaccessible to the researchers. Also excluded were the records of individuals who only attended the initial screening without subsequent return for treatment, and the records of individuals who were assessed but did not need rehabilitation. Initially, 476 records were identified, of which 78 were excluded due to incomplete data, resulting in a final sample of 398 individuals. This was a convenience sample, composed of all available and eligible records.

### Variables 

The following variables were collected, all of which were categorized as presented in the results tables.

- Sociodemographic: sex (male, female), age group (in years: 18-39, 40-59, ≥60), marital status, schooling and work situation.
- Clinical: presence of comorbidities (hypertension, diabetes, obesity, heart diseases, lung diseases), post-COVID symptoms (dyspnea, fatigue, musculoskeletal pain, cognitive changes), prior need for hospitalization (yes, no), length of inpatient stay (in days) and use of oxygen inhalation therapy (yes, no).
- Outcomes of interest: hospital discharge, for service users who completed program follow-up and received formal discharge registered on their medical records; and dropout, for service users who interrupted follow-up before completing it, either due to non-attendance at appointments or withdrawal.

### Data sources and measurement

The information was obtained directly from the program’s electronic medical records, using a standardized instrument developed from the literature and previously tested to ensure uniform data collection.

### Possible biases

The possibility of selection bias was acknowledged, since the data referred only to users with follow-up in a single program, which may limit the generalizability of the findings. Furthermore, information bias may have occurred due to incomplete or inconsistent medical records. In order to minimize these risks, only medical records with complete information on the key variables were included.

### Study size

A sample size calculation was not performed, since all available and eligible medical records were included (convenience sample).

### Statistical analysis 

The collected data were organized on a Microsoft Excel spreadsheet in (version 2018) (8), categorized according to sociodemographic, clinical, hospitalization and care characteristics and treatment outcome. Post-COVID-19 symptoms were classified as respiratory, cardiovascular, gastrointestinal, neurological, psychological, musculoskeletal and other (9).

Jamovi statistical software was used for data analysis. Descriptive analyses were performed using absolute and relative frequencies, measures of central tendency and dispersion. In order to assess associations between independent variables (sociodemographic and clinical) and the outcomes of interest (hospital discharge and dropout), prevalence ratios (PR) with respective 95% confidence intervals (95%CI) were calculated. A significance level of p-value<0.05 was adopted. Multivariate analyses were not conducted due to the small sample size and the exploratory nature of the study.

## Results 

The results encompassed the characterization of individuals with post-COVID-19 conditions and a description of the symptoms presented, based on the medical records of 398 individuals treated at the SuperAR Post-COVID Rehabilitation Center and eligible to participate in the study.

The average age of the service users was 48.7 years (median 48.0; standard deviation 14.1), with the 18-59 age group being the most prevalent. Higher frequencies were observed for female participants (55.8%), married participants (54.7%), and those with schooling up to high school education (42.9%). The main comorbidities were sinusitis (44.1%), hypertension (40.9%) and diabetes (23.3%) (Table 1).

**Table 1:** Descriptive analysis of the sociodemographic characteristics (sex, marital status, education level and age group) and clinical characteristics (comorbidities, smoking and alcohol consumption) of people treated at the SuperAR Post-COVID Rehabilitation Center. Campina Grande, 2024 (n=398)

Variables	n (%)
Sex	
Male	184 (46.2)
Female	214 (55.8)
**Marital status^a^**	
Married	152 (54.7)
Stable union	16 (5.8)
Divorced	34 (12.2)
Single	65 (23.4)
Widow	11 (4.0)
**Sch^o^olinga**	
Illiterate	3 (1.9)
Elementary education	35 (22.7)
High school education	66 (42.9)
Higher education	46 (29.9)
Postgraduate	4 (2.6)
**Age group** (years)	
<18	1 (0.3)
18-59	298 (76.4)
≥60	91 (23.3)
Comorbidities^a^	
Asthma	46 (18.6)
Bronchitis	22 (8.9)
Bronchiecstasis	5 (2.0)
Diabetes	60 (23.3)
Hypertension	101 (40.9)
Sinusitis	109 (44.1)
Rhinitis	14 (5.7)
Other	25 (10.0)
**Tobacco smoking**	
No	250 (80.4)
Yes	61 (19.6)
**Alcohol use**	
No	277 (88.8)
Yes	35 (11.2)

^a^Missing data: marital status (n=120), schooling (n=244), age (n=8) and comorbidities (n=151).

Most people with post-COVID conditions were hospitalized during the infection period (53.8%), in hospital wards (70.0%) and required oxygen inhalation therapy (41.8%). Average length of stay was 11.3 days (median 8; standard deviation 10.4). A predominant proportion of program users received respiratory physiotherapy (72.1%), followed by motor therapy (25.4%). Of the 398 medical records analyzed, 282 service users (78.1%) were discharged from the Rehabilitation Center and 79 (21.9%) dropped out of treatment (Table 2).

**Table 2:** Descriptive analysis of the characteristics of prior hospitalization for COVID-19 and the care received during treatment of individuals treated at the SuperAR Post-COVID Rehabilitation Center. Campina Grande, 2024 (n=398)

Variables	n (%)
**Hospitalization during COVID-19 infection^a^**	
Yes	163 (53.8)
No	140 (46.2)
**Hospitalization unit^a^**	
Hospital ward	105 (70.0)
Intensive care unit	45 (30.0)
**Length of inpatient stay** (days)^a^	
1-15	134 (76.6)
16-30	32 (18.3)
>30	9 (5.1)
**Oxygen inhalation therapy during hospitalization**	
No	177 (58.2)
Yes	127 (41.8)
**Therapies performed at the SuperAR**	
Respiratory physiotherapy	287 (72.1)
Motor therapy	101 (25.4)
Acupuncture	41 (10.3)
Neurology	9 (2.3)
**Treatment outcome at the SuperAR**	
Dropout	79 (21.9)
Hospital discharge	282 (78.1)

^a^Missing data: hospitalization (n=95), hospitalization unit (n=13) and length of inpatient stay (n=33).

**Figure 1:**
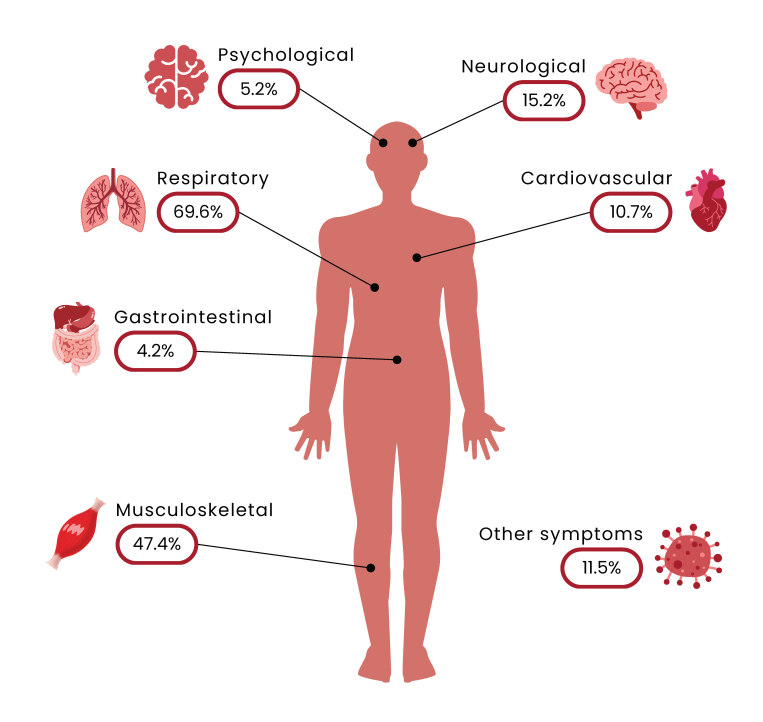
Distribution of post-COVID-19 symptoms (psychological, neurological, respiratory, cardiovascular, gastrointestinal, musculoskeletal and other symptoms) by body system of people treated at the SuperAR Post-COVID Rehabilitation Center. Campina Grande, 2024 (n=398)

Analysis of post-COVID-19 conditions showed that people treated at the SuperAR Post-COVID Rehabilitation Center had a higher prevalence of respiratory symptoms (69.6%) (Figure 1).

Regarding association between sociodemographic and clinical characteristics and treatment outcome (hospital discharge or dropout), significance was observed for sex, occurrence of hospitalization, and provision of oxygen inhalation therapy during hospitalization. Women presented a higher proportion of dropout compared to men (PR 1.68; 95%CI 1.11; 2.53; p-value 0.012). On the other hand, hospitalized individuals presented lower risk of treatment dropout (PR 0.48; 95%CI 0.31; 0.74; p-value 0.001). Those who received oxygen inhalation therapy also had lower risk of dropout (PR 0.50; 95%CI 0.31; 0.82; p-value 0.004) (Table 3).

**Table 3:** Unadjusted prevalence ratios (PR) and 95% confidence intervals (95%CI) of association between sociodemographic characteristics and treatment outcome (dropout versus hospital discharge) of people treated at the SuperAR Post-COVID Rehabilitation Center. Campina Grande, 2024 (n=361)

Variables	Treatment outcome		p-value	PR (95%CI)
	Dropout	Hospital discharge		
	n (%)	n (%)		
Sex			0.012	
Male	28 (16.2)	145(83.8)		
Female	51 (27.1)	137 (79.9)		1.68 (1.11; 2.53)
**Marital status^a^**			0.749^b^	
Married	38 (26.0)	108 (74.0)		
Stable union	2 (12.5)	14 (87.5)		
Divorced	6 (19.4)	25 (80.6)		
Single	14 (22.2)	49 (77.8)		
Widow	2 (25.0)	6 (75.0)		
Schooling^a^			0.736	
Illiterate	-	3 (100.0)		
Elementary education	6 (17.1)	29 (82.9)		
High school education	16 (26.2)	45 (73.8)		
Higher education	11 (24.4)	34 (75.6)		
Postgraduate	1 (25.0)	3 (75.0)		
**Tobacco smoking^a^**			0.554	
No	58 (24.4)	180 (75.6)		
Yes	12 (20.7)	46 (79.3)		
**Alcohol use ^a^**			0.596	
No	63 (24.0)	199 (76.0)		
Yes	7 (20.0)	28 (80.0)		
Hospitalization^a^			<0.001	
Yes	25 (16.2)	129 (83.8)		0.48 (0.31; 0.74)
No	45 (33.8)	88 (66.2)		
**Hospitalization unit ^a^**			0.690	
Hospital ward	15 (15.2)	84 (84.8)		
Intensive care unit	8 (17.8)	37 (82.2)		
**Oxygen inhalation therapy during hospitalization^a^**			0.004	
No	49 (29.2)	119 (70.8)		
Yes	18 (14.6)	105 (85.4)		0.50 (0.31; 0.82)
**Respiratory symptoms**			0.052	
Yes	63 (24.6)	193 (75.4)		
No	14 (14.9)	80 (85.1)		
**Cardiovascular symptoms**			0.131	
Yes	12 (31.6)	26 (68.4)		
No	65 (20.8)	247 (79.2)		
**Gastrointestinal symptoms**			0.650	
Yes	2 (16.7)	10 (83.3)		
No	75 (22.2)	263 (77.8)		
**Neurological symptoms**			0.097	
Yes	62 (20.8)	236 (79.2)		
No	14 (27.5)	37 (72.5)		
**Psychological symptoms^a^**			0.517	
Yes	3 (30.0)	7 (70.0)		
No	73 (21.6)	265 (78.4)		
**Musculoskeletal symptoms^a^**			0.311	
Yes	40 (24.4)	124 (75.6)		
No	37 (19.9)	149 (80.1)		
**Other symptoms^a^**			0.881	
Yes	8 (21.1)	30 (78.9)		
No	69 (22.1)	243 (77.9)		

^a^Missing data: marital status (n=97), schooling (n=213), tobacco smoking (n=65), alcohol use (n=64), hospitalization (n=74), hospitalization unit n=217), oxygen inhalation therapy (n=90), respiratory symptoms (n=11), cardiovascular symptoms (n=258), gastrointestinal symptoms (n=86), neurological symptoms (n=12), psychological symptoms (n=13), musculoskeletal symptoms (n=11) and other symptoms (n=11); ^b^Fisher’s exact test.

When analyzing association between pre-existing comorbidities and treatment outcome, it was found that presence of bronchiectasis was associated with higher prevalence of treatment dropout (PR 2.05; 95%CI 1.34; 3.12; p-value 0.004), since all individuals with this condition discontinued follow-up (Table 4).

**Table 4:** Unadjusted prevalence ratios (PR) and 95% confidence intervals (95%CI) of association between comorbidities and treatment outcome (dropout versus hospital discharge) of people treated at the SuperAR Post-COVID Rehabilitation Center. Campina Grande, 2024 (n=236)

Variables	Treatment outcome		p-value	PR (95%CI)
	Dropout	Hospital discharge		
	n (%)	n (%)		
Bronchitis			0.796	
No	54 (25.2)	160 (74.8)		
Yes	5 (22.7)	17 (77.3)		
Asthma			0.503	
No	46 (24.1)	145 (75.9)		
Yes	13 (28.9)	32 (71.1)		
Tuberculosis			0.244	
No	57 (24.6)	175 (75.4)		
Yes	2 (50.0)	2 (50.0)		
Sinusitis			0.940	
No	33 (24.8)	100 (75.2)		
Yes	26 (25.2)	77 (78.4)		
**Chronic obstructive pulmonary disease**			0.268	
No	56 (24.5)	173 (75.5)		
Yes	3 (42.9)	4 (57.1)		
**Plural effusion**			1.000^a^	
No	59 (25.2)	175 (74.8)		
Yes	-	2 (100.0)		
Bronchiecstasis			0.004^a^	
No	55 (23.7)	177 (76.3)		
Yes	4 (100.0)	-		2.05 (1.34; 3.12)
Diabetes			0.600	
No	46 (25.8)	132 (74.2)		
Yes	13 (22.4)	45 (77.6)		
**Hypertension**			0.702	
No	34 (24.1)	107 (75.9)		
Yes	25 (26.3)	70 (73.7)		
Rhinitis			0.340	
No	54 (24.3)	168 (75.7)		
Yes	5 (35.7)	9 (64.3)		
**Heart disease**			0.825	
No	57 (24.9)	172 (75.1)		
Yes	2 (28.6)	5 (71.4)		
Fibromyalgia			0.068	
No	56 (24.2)	175 (75.8)		
Yes	3 (60.0)	2 (40.0)		

^a^Fisher’s exact test.

## Discussion 

This study characterized the profile of people with post-COVID-19 conditions and factors associated with treatment outcomes. Post-COVID clinical conditions were treated at a rehabilitation center in Campina Grande, between January 2021 and December 2023. This period was considered to be one of intense demand for specialized services, marked by the need to gain a better understanding of the prolonged manifestations of the disease and to implement effective therapeutic strategies for functional recovery and improved quality of life of these individuals.

Post-COVID clinical conditions manifest themselves in approximately 10% to 20% of people who develop the disease and can have short- and long-term effects (10). We identified that most service users included in our study sample presented respiratory symptoms, followed by musculoskeletal symptoms.

Service users who experienced physiological changes such as muscle mass loss during the hospitalization period did not fully recover after six months and showed decreased strength (11). This result justifies the findings of this study. Among the symptoms presented by the individuals were arthralgia and dyspnea, which persisted for a period exceeding 12 months after COVID-19 infection. This was consistent with a setting involving 204 people in Fortaleza in 2021 (12).

The disproportionate impact of COVID-19 on women attending the SuperAR Center was identified in the sample characterization. It was observed that they represented the majority of cases followed during the period, revealing higher prevalence of persistent symptoms and greater demand for specialized rehabilitation care.

The pandemic exacerbated health-related gender inequality and particularly affected women. They not only make up the majority of health professionals, but are also frequently primary caregivers in domestic and community settings (13). Furthermore, evidence has indicated that women are more susceptible to long COVID (14-16).

In addition to women’s greater exposure to illness, they were also the ones who most frequently dropped out of treatment (PR 1.52; 95%CI 1.09; 2.11; p-value 0.012). There is no single explanation for the higher dropout rate among women, but there are sociocultural aspects that may lead women to discontinue treatment, such as high domestic demands and working outside the home.

The practical implications of providing care to women after COVID-19 infection must be addressed holistically, considering the physical repercussions of the disease, such as persistent fatigue, respiratory and cardiovascular changes, and psychological and social impacts resulting from the illness. In this context, it is possible to reduce treatment dropout by ensuring that these women receive support and that health professionals are attentive to their needs.

Regarding the severity of post-COVID-19 symptoms in adults, demographic factors such as advanced age, male sex, ethnicity and pre-existing comorbidities are considered risk factors for the development and severity of COVID-19 (17).

Regarding comorbidities identified in the study population, bronchiectasis was the only disease that had a significant association with treatment dropout (p-value 0.004). This finding may be related to the chronic course of the disease and the demands of prolonged and complex treatment, which requires a daily routine of care and, consequently, may hinder continuous adherence. A significant proportion of infected individuals, even after treatment, present prolonged symptoms, characterized by persistent lung damage and alterations in respiratory function, which may remain for months after the acute phase of the disease (18).

Post-COVID symptoms have highlighted the need for the implementation of medium- and long-term rehabilitation actions by multidisciplinary teams. Therefore, it is essential to provide comprehensive, individualized and continuous care focused on physical recovery and social and occupational reintegration, contributing to reducing long-term effects and promoting the overall well-being of service users.

There is high prevalence of persistent symptoms among people affected by Post-COVID syndrome. However, this condition remains poorly recognized in health services, which hinders timely service user access to necessary care. There is also low awareness and understanding of long COVID, both among health professionals and in society in general (19).

An integrated and multidisciplinary care approach in the management of post-COVID-19 syndrome should include detailed clinical assessment in order to ensure effective treatment of both pre-existing comorbidities and the symptoms of post-COVID-19 syndrome (20). Identification of new emerging health conditions in the post-COVID-19 context should be carefully monitored to ensure full recovery and prevent further complications.

The predominance of respiratory complications can be attributed to the mode of transmission and primary location of the virus in the respiratory tract, which can lead to more severe conditions such as acute respiratory distress syndrome and hypoxemia (21). In this context, use of oxygen inhalation therapy during hospitalization (p-value 0.004) demonstrated statistically significant association with treatment outcome. A higher proportion of individuals who did not use oxygen inhalation therapy dropped out of treatment compared to those who did. On the other hand, respiratory physiotherapy emerged as the most frequently applied therapy, followed by motor therapy.

Treatment dropout can be minimized through care coordination in primary health care, which is fundamental to ensuring effective follow-up of people with post-COVID-19 symptoms, as it promotes integration of actions at different levels of care. This coordinated approach involves various health professionals who use specific mechanisms to plan care, define flows and facilitate the exchange of information about service users. This is crucial not only for monitoring the progression of symptoms and associated complications, but also for ensuring a smooth transition from acute care to rehabilitation services, increasing the chances of long-term recovery (22,23).

A limitation of this study is that it only analyzed the SuperAR Program, not including other health services that comprise the healthcare networks in Campina Grande. Challenges arose from insufficient information held on medical records, characterized by gaps in the data collected, as several records lacked sufficient information to enable analysis. Additionally, it was not possible to verify the effective coordination of care by primary healthcare. These limitations indicate the need for more comprehensive and detailed future research.

Finally, this study contributed to identifying the characteristics of people with post-COVID-19 conditions and the rehabilitation care actions developed in a large municipality in Northeastern Brazil. Most people with these conditions presented respiratory symptoms and pre-existing comorbidities, such as bronchiectasis, and received physiotherapy and oxygen inhalation therapy. Furthermore, there was greater impact and a higher treatment dropout rate among women.

We highlight the importance of providing a comprehensive approach that considers clinical and social factors in order to improve the organization of healthcare networks in addressing these conditions. The findings reinforce the need for continuity of care and implementation of strategies aligned with the realities of health service territories, promoting improved quality of health and rehabilitation for these individuals.

## Data Availability

All the data supporting the results of this study has been made available on Figshare and can be accessed at: https://figshare.com/s/97a38dc6c78bc33f4fa9.
