# Supplementary material for: Factors associated with post-COVID-19 health conditions at a rehabilitation center: a cross-sectional study, Paraíba, Brazil, 2024
Source: Epidemiol Serv Saude. 2026 Apr 20;35:e20250305. doi: 10.1590/S2237-96222026v35e20250305.en (PMC13102382; doi:10.1590/S2237-96222026v35e20250305.en)
Supplement: Translation

# Translation: Fatores associados às condições de saúde pós-covid-19 em centro de reabilitação: estudo transversal, Paraíba, 2024

**DOI:** 10.1590/S2237-96222026v35e20250305.pt

**Authors:** Karla Karolline Barreto Cardins, Ísis de Siqueira Silva, Amanda Soares, Lara Maria Alves de Carvalho, José Mateus Bezerra da Graça, Cláudia Helena Soares de Morais Freitas

**Affiliations:** 1 Universidade Federal de Campina Grande, Unidade Acadêmica de Enfermagem, Cajazeiras, PB, Brasil; 2 Universidade Federal do Rio Grande do Norte, Natal, RN, Brasil; 3 Universidade Federal do Rio Grande do Norte, Faculdade de Ciências da Saúde do Trairi, Trairi, RN, Brasil; 4 Universidade Federal do Rio Grande do Norte, Programa de Pós-Graduação em Saúde Coletiva, Santa Cruz, RN, Brasil; 5 Universidade Federal da Paraíba, Departamento de Clínica e Odontologia Social, João Pessoa, PB, Brasil

**Keywords:** Síndrome de Pós-Covid-19 Aguda, Atenção à Saúde, Serviços de Reabilitação, Sinais e Sintomas, Estudos Transversais

## Abstract

**Objetivo:**

Caracterizar o perfil das pessoas com condições pós-covid-19 e os fatores associados ao desfecho do tratamento em um centro de reabilitação em Campina Grande, Paraíba.

**Métodos:**

Estudo transversal, com base na análise de 398 prontuários de usuários atendidos em um centro de reabilitação pós-covid. Foram avaliadas variáveis sociodemográficas, clínicas e assistenciais, utilizando-se razão de prevalência (RP) com intervalo de confiança de 95% (IC95%) e nível de significância de 5%. O desfecho de interesse foi o resultado do tratamento, categorizado como alta hospitalar ou abandono.

**Resultados:**

A análise dos 398 prontuários revelou a predominância do sexo feminino (62,6%) e da faixa etária adulta de 18-59 anos (63,3%). O abandono do tratamento foi mais frequente entre mulheres do que entre homens (RP 1,68; IC95% 1,11; 2,53; p-valor 0,012). Pessoas internadas apresentaram menor risco de abandono (RP 0,48; IC95% 0,31; 0,74; p-valor 0,001), e aquelas que receberam oxigenoterapia também tiveram menor risco de abandonar o tratamento (RP 0,50; IC95% 0,31; 0,82; p-valor 0,004). Dos 398 usuários analisados, 282 (78,1%) receberam alta hospitalar e 79 (21,9%) abandonaram o tratamento.

**Conclusão:**

O sexo feminino esteve associado a maior abandono, enquanto a internação e a oxigenoterapia funcionaram como fatores de proteção para a adesão. Esses achados reforçam a necessidade de estratégias de acompanhamento direcionadas, capazes de reduzir perdas no seguimento e promover maior efetividade na reabilitação pós-covid-19 no Sistema Único de Saúde.

**Aspectos éticos**

Esta pesquisa respeitou os princípios éticos, obtendo os seguintes dados de aprovação: :

Comitê de ética em pesquisa: Universidade Estadual da Paraíba

Número do parecer: 6.180.794

Data de aprovação: 13/7/2023

Certificado de apresentação de apreciação ética: 69833323.3.0000.5187

Registro do consentimento livre e esclarecido: Dispensado.

## Introdução 

Em janeiro de 2020, a Organização Mundial da Saúde reconheceu a covid-19 como uma emergência em saúde pública, diante da rápida disseminação global do SARS-CoV-2 (1). A infecção pode variar desde quadros assintomáticos e leves até formas moderadas, graves e críticas, que exigem hospitalização (1). Consequentemente, a pandemia gerou sobrecarga nos sistemas de saúde e ocasionou aumento da morbimortalidade, além de repercussões significativas na saúde mental da população, associadas ao estresse, ao isolamento e às incertezas sociais (2).

Mesmo após a fase aguda da doença, uma parcela das pessoas permanecia sintomática por semanas ou meses. Diante disso, a Organização Mundial da Saúde passou a adotar o termo “síndrome pós-covid”, caracterizado pela persistência de sintomas por mais de três meses, sem explicação por outras condições prévias (3). De forma complementar, o Ministério da Saúde do Brasil empregou a denominação “condições pós-covid-19”, definida como sinais, sintomas e/ou condições que persistem ou surgem após quatro semanas da infecção inicial, podendo evoluir de maneira flutuante, com agravamentos, recidivas ou complicações graves, inclusive meses após a infecção (1).

Esse quadro emergente impôs novos desafios às redes de atenção à saúde e exigiu a reorganização de serviços e estratégias de cuidado. Em dezembro de 2021, o Sistema Único de Saúde incorporou procedimentos específicos para a reabilitação pós-covid, financiados pelo Fundo de Ações Estratégicas e Compensação (4). Todavia, diante da ausência de respostas federais consistentes, diversos estados e municípios assumiram o protagonismo, criando iniciativas próprias de tratamento e reabilitação (5,6).

A atenção primária à saúde tem desempenhado papel central na avaliação e no manejo inicial das pessoas com condições pós-covid, articulando fluxos de referência e contrarreferência com as redes de atenção à saúde. Alguns municípios organizaram serviços especializados de reabilitação para atender a essa demanda crescente (7). Dessa maneira, torna-se essencial conhecer o perfil das pessoas que buscam esses serviços e os fatores associados à evolução do tratamento, uma vez que tal conhecimento pode subsidiar a organização da assistência, otimizar recursos, orientar práticas clínicas baseadas em evidências e fortalecer políticas públicas.

Este estudo teve como objetivo caracterizar o perfil das pessoas com condições pós-covid-19, entre janeiro de 2021 e dezembro de 2023, e os fatores associados ao desfecho do tratamento em um centro de reabilitação de Campina Grande, Paraíba.

## Métodos 

### Delineamento e contexto

Trata-se de estudo transversal, desenvolvido a partir da análise de prontuários eletrônicos de pessoas atendidas pelo Programa SuperAR, vinculado à Prefeitura Municipal de Campina Grande, Paraíba. À época do estudo, o serviço tinha abrangência regional e recebia pessoas encaminhadas e por demanda espontânea. O atendimento era via Sistema Único de Saúde. O período de coleta de dados compreendeu os meses de setembro a dezembro de 2023.

### Participantes e critérios de elegibilidade

Os dados para esta pesquisa provieram dos prontuários de usuários atendidos pelo programa no Centro de Reabilitação Pós-covid SuperAR. Foram incluídos os prontuários das pessoas que receberam assistência no âmbito do Programa SuperAR com diagnóstico confirmado de infecção prévia por covid-19, com os registros acessíveis referentes a pessoas que receberam tratamento (fisioterapia respiratória, motora, acupuntura e neurologia) para os sintomas pós-covid-19 entre janeiro de 2021 e dezembro de 2023. 

Foram excluídos os registros de atendimento psicológico, uma vez que estavam arquivados em um local distinto, não sendo permitido o acesso dos pesquisadores. Também foram excluídos os prontuários das pessoas que apenas compareceram à triagem inicial, sem retorno subsequente para tratamento, e os prontuários das pessoas avaliadas, mas que não apresentavam necessidade de reabilitação. Inicialmente, identificaram-se 476 prontuários, dos quais 78 foram excluídos por conterem dados incompletos, o que totalizou a amostra final de 398 pessoas. Tratou-se de uma amostra de conveniência, composta por todos os registros disponíveis e elegíveis.

### Variáveis 

As variáveis coletadas foram as elencadas a seguir. Todas foram categorizadas conforme apresentado nas tabelas de resultados.

- Sociodemográficas: sexo (masculino, feminino), faixa etária (em anos: 18-39, 40-59, ≥60), estado civil, escolaridade e situação laboral.
- Clínicas: presença de comorbidades (hipertensão, diabetes, obesidade, cardiopatias, pneumopatias), sintomas pós-covid (dispneia, fadiga, dor musculoesquelética, alterações cognitivas), necessidade de hospitalização prévia (sim, não), tempo de internação (em dias) e uso de oxigenoterapia (sim, não).
- Desfechos de interesse: alta hospitalar, para o usuário que completou o acompanhamento no programa e que recebeu registro formal de liberação no prontuário; e abandono, para o usuário que interrompeu o acompanhamento antes da conclusão, seja por não comparecimento às consultas, seja por desistência.

### Fonte de dados e mensuração

As informações foram obtidas diretamente dos prontuários eletrônicos do programa, por meio de instrumento padronizado, elaborado a partir da literatura e testado previamente para uniformizar a coleta.

### Possíveis vieses

Reconheceu-se a possibilidade de viés de seleção, uma vez que os dados se referiram apenas a usuários acompanhados em um único programa, o que pode limitar a generalização dos achados. Além disso, pode ter ocorrido viés de informação decorrente de registros incompletos ou inconsistentes nos prontuários. Para minimizar esses riscos, foram incluídos apenas prontuários com informações completas nas variáveis-chave.

### Tamanho do estudo

Não foi realizado cálculo amostral, visto que todos os prontuários disponíveis e elegíveis foram incluídos (amostra de conveniência).

### Análise estatística

Os dados coletados foram organizados em uma planilha no software Microsoft Excel (versão 2108) (8), categorizados conforme as características sociodemográficas, clínicas, da internação e assistência realizada e o desfecho do tratamento. Os sintomas pós-covid-19 foram classificados como respiratórios, cardiovasculares, gastrointestinais, neurológicos, psicológicos, musculoesqueléticos e outros (9).

O software estatístico Jamovi foi utilizado para a análise dos dados. Análises descritivas foram realizadas por meio de frequências absolutas e relativas, medidas de tendência central e dispersão. Para avaliar associações entre variáveis independentes (sociodemográficas e clínicas) e os desfechos de interesse (alta hospitalar e abandono), foram calculadas razões de prevalência (RP) com respectivos intervalos de confiança de 95% (IC95%). O nível de significância adotado foi de p-valor<0,05. Não foram conduzidas análises multivariadas devido ao tamanho reduzido da amostra e ao caráter exploratório do estudo.

## Resultados 

Os resultados englobaram a caracterização das pessoas com condições pós-covid-19 e a descrição dos sintomas apresentados. Os prontuários de 398 pessoas atendidas no Centro de Reabilitação Pós-covid SuperAR foram considerados elegíveis a participar do estudo. 

A idade média dos usuários foi 48,7 anos (mediana 48,0; desvio-padrão 14,1), sendo a faixa etária 18-59 anos a mais prevalente. Foram observadas maiores frequências para participantes do sexo feminino (55,8%), casados (54,7%), com nível de escolaridade até o ensino médio (42,9%). As principais comorbidades foram sinusite (44,1%), hipertensão (40,9%) e diabetes (23,3%) (Tabela 1).

**Tabela 1:** Análise descritiva das características sociodemográficas (sexo, estado civil, escolaridade e faixa etária) e clínicas (comorbidades, tabagismo e etilismo) de pessoas atendidas no Centro de Reabilitação Pós-covid SuperAR. Campina Grande, 2024 (n=398)

Variáveis	n (%)
Sexo	
Masculino	184 (46,2)
Feminino	214 (55,8)
**Estado civil^a^**	
Casado	152 (54,7)
União estável	16 (5,8)
Divorciado	34 (12,2)
Solteiro	65 (23,4)
Viúvo	11 (4,0)
**Escola^r^idadea**	
Não alfabetizado	3 (1,9)
Ensino fundamental	35 (22,7)
Ensino médio	66 (42,9)
Ensino superior	46 (29,9)
Pós-graduação	4 (2,6)
**Faixa ^e^táriaa** (anos)	
<18	1 (0,3)
18-59	298 (76,4)
≥60	91 (23,3)
Comorbidades^a^	
Asma	46 (18,6)
Bronquite	22 (8,9)
Bronquiectasia	5 (2,0)
Diabetes	60 (23,3)
Hipertensão	101 (40,9)
Sinusite	109 (44,1)
Rinite	14 (5,7)
Outras	25 (10,0)
Tabagismo	
Não	250 (80,4)
Sim	61 (19,6)
Etilismo	
Não	277 (88,8)
Sim	35 (11,2)

^a^Dados ausentes: estado civil (n=120), escolaridade (n=244), idade (n=8) e comorbidades (n=151).

A maioria das pessoas com condições pós-covid esteve internada durante o período de infecção (53,8%), em enfermarias (70,0%), e necessitou de oxigenoterapia (41,8%). O tempo médio de internação foi 11,3 dias (mediana 8; desvio-padrão 10,4). A parcela predominante dos usuários do programa recebeu fisioterapia respiratória (72,1%), seguida por fisioterapia motora (25,4%). Dos 398 prontuários analisados, 282 usuários (78,1%) receberam alta hospitalar e 79 (21,9%) abandonaram o tratamento (21,9%) (Tabela 2).

**Tabela 2:** Análise descritiva das características de internação prévia por covid-19 e da assistência recebida durante o tratamento das pessoas atendidas no Centro de Reabilitação Pós-covid SuperAR. Campina Grande, 2024 (n=398)

Variáveis	n (%)
**Internação durante infecção por covid-19^a^**	
Sim	163 (53,8)
Não	140 (46,2)
**Unidade em que esteve internada^a^**	
Enfermaria	105 (70,0)
Unidade de terapia intensiva	45 (30,0)
**Tempo de internação** (dias)^a^	
1-15	134 (76,6)
16-30	32 (18,3)
>30	9 (5,1)
**Oxigenoterapia durante internação**	
Não	177 (58,2)
Sim	127 (41,8)
**Terapias realizadas no SuperAR**	
Fisioterapia respiratória	287 (72,1)
Fisioterapia motora	101 (25,4)
Acupuntura	41 (10,3)
Neurologista	9 (2,3)
**Conclusão do tratamento no SuperAR**	
Abandono	79 (21,9)
Alta hospitalar	282 (78,1)

^a^Dados ausentes: internação (n=95), unidade de internação (n=13) e tempo de internação (n=33).

O levantamento das condições pós-covid-19 evidenciou que as pessoas atendidas no Centro de Reabilitação Pós-covid SuperAR apresentaram maior prevalência de sintomas respiratórios (69,6%) (Figura 1).

**Figura 1:** Distribuição dos sintomas pós-covid-19 (psicológicos, neurológicos, respiratórios, cardiovasculares, gastrointestinais, musculoesqueléticos e outros) por sistema corporal das pessoas atendidas no Centro de Reabilitação Pós-covid SuperAR. Campina Grande, 2024 (n=398)

Quanto à associação entre características sociodemográficas e clínicas e o desfecho do tratamento (alta hospitalar ou abandono), observou-se significância para o sexo, para a ocorrência de internação e para a oferta de oxigenoterapia durante a hospitalização. As mulheres apresentaram maior proporção de abandono em comparação aos homens (RP 1,68; IC95% 1,11; 2,53; p-valor 0,012). Por outro lado, pessoas internadas apresentaram menor risco de abandono do tratamento (RP 0,48; IC95% 0,31; 0,74; p-valor 0,001). Aqueles que receberam oxigenoterapia também tiveram menor risco de abandono (RP 0,50; IC95% 0,31; 0,82; p-valor 0,004) (Tabela 3).

**Tabela 3:** Razões de prevalências (RP) e intervalos de confiança de 95% (IC95%) brutos da associação entre características sociodemográficas e desfecho do tratamento (abandono versus alta hospitalar) de pessoas atendidas no Centro de Reabilitação Pós-covid SuperAR. Campina Grande, 2024 (n=361)

Variáveis	Desfecho do tratamento		p-valor	RP (IC95%)
	Abandono	Alta hospitalar		
	n (%)	n (%)		
Sexo			0,012	
Masculino	28 (16,2)	145(83,8)		
Feminino	51 (27,1)	137 (79,9)		1,68 (1,11; 2,53)
**Estado civil^a^**			0,749^b^	
Casado	38 (26,0)	108 (74,0)		
União estável	2 (12,5)	14 (87,5)		
Divorciado	6 (19,4)	25 (80,6)		
Solteiro	14 (22,2)	49 (77,8)		
Viúvo	2 (25,0)	6 (75,0)		
Escolaridade^a^			0,736	
Não alfabetizado	-	3 (100,0)		
Ensino fundamental	6 (17,1)	29 (82,9)		
Ensino médio	16 (26,2)	45 (73,8)		
Ensino superior	11 (24,4)	34 (75,6)		
Pós-graduação	1 (25,0)	3 (75,0)		
Tabagismo^a^			0,554	
Não	58 (24,4)	180 (75,6)		
Sim	12 (20,7)	46 (79,3)		
Etilismo^a^			0,596	
Não	63 (24,0)	199 (76,0)		
Sim	7 (20,0)	28 (80,0)		
**Houve internação^a^**			<0,001	
Sim	25 (16,2)	129 (83,8)		0,48 (0,31; 0,74)
Não	45 (33,8)	88 (66,2)		
**Unidade em que esteve internada^a^**			0,690	
Enfermaria	15 (15,2)	84 (84,8)		
Unidade de terapia intensiva	8 (17,8)	37 (82,2)		
**Oxigenoterapia durante internação^a^**			0,004	
Não	49 (29,2)	119 (70,8)		
Sim	18 (14,6)	105 (85,4)		0,50 (0,31; 0,82)
**Sintomas respiratórios^a^**			0,052	
Sim	63 (24,6)	193 (75,4)		
Não	14 (14,9)	80 (85,1)		
**Sintomas cardiovasculares^a^**			0,131	
Sim	12 (31,6)	26 (68,4)		
Não	65 (20,8)	247 (79,2)		
**Sintomas gastrointestinais^a^**			0,650	
Sim	2 (16,7)	10 (83,3)		
Não	75 (22,2)	263 (77,8)		
**Sintomas neurológicos^a^**			0,097	
Sim	62 (20,8)	236 (79,2)		
Não	14 (27,5)	37 (72,5)		
**Sintomas psicológicos^a^**			0,517	
Sim	3 (30,0)	7 (70,0)		
Não	73 (21,6)	265 (78,4)		
**Sintomas musculoesqueléticos^a^**			0,311	
Sim	40 (24,4)	124 (75,6)		
Não	37 (19,9)	149 (80,1)		
**Outros sintomas^a^**			0,881	
Sim	8 (21,1)	30 (78,9)		
Não	69 (22,1)	243 (77,9)		

^a^Dados ausentes: estado civil (n=97), escolaridade (n=213), tabagismo (n=65), etilismo (n=64), internação (n=74), unidade de internação (n=217), oxigenoterapia (n=90), sintomas respiratórios (n=11), sintomas cardiovasculares (n=258), sintomas gastrointestinais (n=86), sintomas neurológicos (n=12), sintomas psicológicos (n=13), sintomas musculoesqueléticos (n=11) e outros sintomas (n=11); ^b^ Teste exato de Fisher.

Na análise da associação entre comorbidades prévias e o desfecho do tratamento, observou-se que a presença de bronquiectasia esteve associada à maior prevalência de abandono do tratamento (RP 2,05; IC95% 1,34; 3,12; p-valor 0,004), uma vez que todos as pessoas com essa condição interromperam o acompanhamento (Tabela 4).

**Tabela 4:** Razões de prevalências (RP) e intervalos de confiança de 95% (IC95%) brutos da associação entre comorbidades e desfecho do tratamento (abandono versus alta hospitalar) de pessoas atendidas no Centro de Reabilitação Pós-covid SuperAR. Campina Grande, 2024 (n=236)

Variáveis	Desfecho do tratamento		p-valor	RP (IC95%)
	Abandono	Alta hospitalar		
	n (%)	n (%)		
Bronquite			0,796	
Não	54 (25,2)	160 (74,8)		
Sim	5 (22,7)	17 (77,3)		
Asma			0,503	
Não	46 (24,1)	145 (75,9)		
Sim	13 (28,9)	32 (71,1)		
Tuberculose			0,244	
Não	57 (24,6)	175 (75,4)		
Sim	2 (50,0)	2 (50,0)		
Sinusite			0,940	
Não	33 (24,8)	100 (75,2)		
Sim	26 (25,2)	77 (78,4)		
**Doença pulmonar obstrutiva crônica**			0,268	
Não	56 (24,5)	173 (75,5)		
Sim	3 (42,9)	4 (57,1)		
**Derrame pleural**			1,000^a^	
Não	59 (25,2)	175 (74,8)		
Sim	-	2 (100,0)		
Bronquiectasia			0,004^a^	
Não	55 (23,7)	177 (76,3)		
Sim	4 (100,0)	-		2,05 (1,34; 3,12)
Diabetes			0,600	
Não	46 (25,8)	132 (74,2)		
Sim	13 (22,4)	45 (77,6)		
**Hipertensão**			0,702	
Não	34 (24,1)	107 (75,9)		
Sim	25 (26,3)	70 (73,7)		
Rinite			0,340	
Não	54 (24,3)	168 (75,7)		
Sim	5 (35,7)	9 (64,3)		
Cardiopatia			0,825	
Não	57 (24,9)	172 (75,1)		
Sim	2 (28,6)	5 (71,4)		
Fibromialgia			0,068	
Não	56 (24,2)	175 (75,8)		
Sim	3 (60,0)	2 (40,0)		

^a^Teste exato de Fisher.

## Discussão 

Este estudo caracterizou o perfil das pessoas com condições pós-covid-19 e fatores associados ao desfecho do tratamento. As condições clínicas pós-covid foram tratadas em um centro de reabilitação em Campina Grande, Paraíba, entre janeiro de 2021 e dezembro de 2023. Esse período foi considerado de intensa demanda por serviços especializados, marcado pela necessidade de compreender melhor as manifestações prolongadas da doença e implementar estratégias terapêuticas eficazes para a recuperação funcional e a melhoria da qualidade de vida das pessoas.

As condições clínicas pós-covid são evidenciadas em cerca de 10% a 20% das pessoas que desenvolvem a doença e podem ter efeitos a curto e longo prazo (10). A amostra deste estudo identificou que a maioria dos usuários apresentou sintomas respiratórios, seguido de sintomas musculoesqueléticos.

Usuários que apresentaram alterações fisiológicas como perda da massa muscular durante o período de internação não se recuperaram totalmente após seis meses e apresentaram diminuição da força (11). Com isso, pode-se justificar os achados deste estudo. Entre os sintomas apresentados pelos indivíduos estavam a artralgia e dispneia, que perduraram por período superior a 12 meses após a infecção pela covid-19. Isso esteve de acordo com um contexto com 204 pessoas em Fortaleza em 2021 (12).

O impacto desproporcional da covid-19 sobre as mulheres atendidas pelo Centro SuperAR foi identificado na caracterização da amostra. Observou-se que elas representaram a maioria dos casos acompanhados no período, revelando maior prevalência de sintomas persistentes e maior procura por atendimento especializado em reabilitação.

A pandemia exacerbou a desigualdade de gênero na saúde e afetou especialmente as mulheres. Estas não apenas compõem a maioria dos profissionais da saúde, mas também são frequentemente as principais cuidadoras em contextos domésticos e comunitários (13). Além disso, evidências indicaram que mulheres são mais suscetíveis à covid longa (14-16).

Somado à maior exposição de mulheres ao adoecimento, também foram essas que em maior proporção abandonaram o tratamento (RP 1,52; IC95% 1,09; 2,11; p-valor 0,012). Não há uma justificativa que direcione o entendimento do maior abandono do tratamento por mulheres, mas existem aspectos socioculturais que podem induzir a mulher a desistir de seguir com um tratamento, como as altas demandas domésticas e o trabalho fora de casa.

As implicações práticas na assistência à mulher pós-infecção de covid-19 devem ser tratadas de forma integral, considerando as repercussões físicas da doença, como fadiga persistente, alterações respiratórias e cardiovasculares, e os impactos psicológicos e sociais decorrentes do adoecimento. Nesse contexto, é possível reduzir as desistências do tratamento garantindo o acolhimento e a escuta das necessidades dessas mulheres.

Em relação à gravidade dos sintomas pós-covid-19, em adultos, fatores demográficos como idade avançada, sexo masculino, etnia e comorbidades preexistentes são considerados fatores de risco para o desenvolvimento e gravidade da covid-19 (17).

Quanto às comorbidades identificadas na população do estudo, a bronquiectasia foi a única doença que teve associação significativa com o desfecho abandono do tratamento (p-valor 0,004). Esse achado pode estar relacionado ao curso crônico da enfermidade e às exigências de um tratamento prolongado e complexo, que demanda uma rotina diária de cuidados e, consequentemente, pode dificultar a adesão contínua. Uma parcela significativa dos indivíduos infectados, mesmo após o tratamento, apresenta prolongamento dos sintomas, caracterizado por lesões pulmonares persistentes e alterações da função respiratória, que podem permanecer por meses após a fase aguda da doença (18).

Os sintomas pós-covid têm alertado para a necessidade da implementação de ações de reabilitação a médio e longo prazo por equipes multidisciplinares. Com isso, torna-se fundamental oferecer um cuidado abrangente, individualizado e contínuo, voltado à recuperação física e à reinserção social e laboral, contribuindo para reduzir as sequelas e promover a melhoria global do bem-estar dos usuários.

A síndrome pós-covid apresenta alta prevalência de sintomas persistentes na população acometida. No entanto, essa condição permanece pouco reconhecida nos serviços de saúde, o que dificulta o acesso oportuno dos usuários aos cuidados necessários. Observa-se, ainda, baixa conscientização e compreensão sobre a covid longa, tanto entre profissionais de saúde quanto na sociedade em geral (19).

A abordagem de cuidado integrado e multiprofissional no manejo da síndrome pós-covid-19 deve incluir uma avaliação clínica detalhada para tratar efetivamente as comorbidades preexistentes em conjunto com os sintomas da síndrome pós-covid-19 (20). A identificação de novas patologias emergentes no contexto pós-covid-19 deve ser cuidadosamente monitorada para garantir a recuperação integral e prevenir complicações adicionais.

A predominância de complicações respiratórias pode ser atribuída ao modo de transmissão e localização primária do vírus nas vias respiratórias, o que pode levar a condições mais graves, como a síndrome respiratória aguda grave e a hipoxemia (21). Diante disso, o uso de oxigenoterapia durante a internação (p-valor 0,004) demonstrou a associação estatisticamente significativa com o desfecho do tratamento. Observou-se que uma proporção maior de indivíduos que não utilizaram oxigenoterapia abandonou o tratamento em comparação com aqueles que fizeram uso dela. Por outro lado, a fisioterapia respiratória emergiu como a terapêutica mais frequentemente aplicada, seguida pela fisioterapia motora. 

As desistências ao tratamento podem ser minimizadas através da coordenação do cuidado pela atenção primária à saúde, fundamental para garantir o acompanhamento efetivo das pessoas com sintomas pós-covid-19, uma vez que promove a integração de ações em diferentes níveis de atenção. Essa abordagem coordenada envolve diversos profissionais de saúde que utilizam mecanismos específicos para planejar a assistência, definir fluxos e facilitar a troca de informações sobre os usuários. Isso é crucial não apenas para monitorar a progressão dos sintomas e das complicações associadas, mas também para garantir a transição suave de cuidados agudos para serviços de reabilitação, aumentando as chances de recuperação a longo prazo (22,23).

Como limitação deste estudo, destaca-se que foi analisado apenas o Programa SuperAR, não contemplando os demais serviços de saúde que compõem as redes de atenção à saúde de Campina Grande, Paraíba. Enfrentaram-se desafios decorrentes da insuficiência de informações nos prontuários, caracterizados por lacunas nos dados coletados, visto que não havia informações suficientes para análise em vários prontuários. Adicionalmente, não foi possível verificar a efetiva coordenação do cuidado pela atenção primária à saúde. Essas limitações indicam a necessidade de pesquisas futuras mais abrangentes e detalhadas.

Por fim, este estudo contribuiu na identificação das características das pessoas com condições pós-covid-19 e das ações assistenciais de reabilitação desenvolvidas em um município de grande porte do Nordeste. As pessoas com essas condições apresentaram, em sua maioria, sintomas respiratórios, comorbidades prévias, como bronquiectasia, e receberam assistência de fisioterapia e oxigenoterapia. Ainda, houve maior impacto e abandono ao tratamento no público de mulheres.

Destaca-se a importância de ofertar uma abordagem integral que considere fatores clínicos e sociais para aprimorar a organização das redes de atenção à saúde no atendimento a essas condições. Os achados reforçam a necessidade da continuidade do cuidado e da implementação de estratégias alinhadas à realidade dos territórios, promovendo a melhoria da qualidade em saúde e a reabilitação dessas pessoas.

## Data Availability

Todo o conjunto de dados que dá suporte aos resultados deste estudo foi disponibilizado no Figshare e pode ser acessado em: https://figshare.com/s/97a38dc6c78bc33f4fa9.
